# Role of percutaneous liver biopsy in infantile cholestasis: cohort from Arabs

**DOI:** 10.1186/s12876-021-01699-4

**Published:** 2021-03-12

**Authors:** Amna Basheer M. Ahmed, Musa Ahmad Fagih, Muhammed Salman Bashir, Abdulrahman Abdullah Al-Hussaini

**Affiliations:** 1 The Division of Pediatric Gastroenterology, Children’s Specialized Hospital, King Fahad Medical City, P. O. Box 59046, Riyadh, Postal Code 11525 Kingdom of Saudi Arabia; 2 Department of Pathology and Laboratory Medicine, King Saud Medical City, Riyadh, Kingdom of Saudi Arabia; 3 Department of Biostatistics, Research Services Administration, King Fahad Medical City, Riyadh, 11525 Kingdom of Saudi Arabia; 4 College of Medicine, Alfaisal University, Riyadh, Kingdom of Saudi Arabia; 5 Prince Abdullah Bin Khalid Celiac Disease Research Chair, Department of Pediatrics, Faculty of Medicine, King Saud University Riyadh, Riyadh, Kingdom of Saudi Arabia

**Keywords:** Liver biopsy, Pathology, Indications, Infant, Saudi Arabia, Cholestasis

## Abstract

**Background:**

Investigators from different parts of the world are calling for a re-evaluation of the role of liver biopsy (LB) in the evaluation of infantile cholestasis (IC), especially in the light of emerging non-invasive diagnostic technologies. Therefore, this retrospective single-center study was conducted to determine the impact of LB on the diagnosis and management of IC in a cohort from Arabs.

**Methods:**

From 2007 until 2019, 533 cases of IC were referred for evaluation. All infants who underwent LB were included in the study. We categorized the yield of LB into: (1) defined specific diagnosis; (2) excluded an important diagnosis. A single pathologist reviewed and made the histology report.

**Results:**

122 LB specimens met the inclusion criteria. The main indication for LB was a high suspicion of biliary atresia (BA) [high gamma-glutamyl transferase (GGT) cholestasis and pale stool] in 46 cases (37.8%). Liver biopsy had sensitivity of 86.4%, specificity (66.7%), PPV (70.4%), NPV (84.2%) in diagnosing BA. LB had a direct impact on clinical management in 52 cases (42.6%): (1) The true diagnosis was suggested by LB in 36 cases; (2) LB excluded BA and avoided intraoperative cholangiogram in 16 cases with high suspicion of BA. Among the 76 cases with low suspicion of BA, LB suggested the true diagnosis or helped to initiate specific management in 8 cases only (10.5%). In contrast, molecular testing confirmed the diagnosis in 48 (63%).

**Conclusion:**

LB continues to be an important tool in the workup of cases with a high suspicion of BA. The low yield of LB in cases with low suspicion of BA calls for a re-evaluation of its role in these cases in whom early incorporation of cholestasis sequencing gene  panels can have a better diagnostic yield.

## Introduction

Histological examination of liver tissue has long been considered the cornerstone of the diagnostic workup of infants with cholestatic jaundice as recommended in both the old and most recent guidelines by the North American (NASPGHAN) and European society of pediatric gastroenterology, hepatology, and nutrition (ESPGHAN) [1, 2]. Infantile cholestasis (IC) is a diagnostic dilemma that present a challenge to pediatric gastroenterologists. The liver in neonates and young infants is vulnerable to insults of different causes and usually responds to injury by manifesting cholestasis with considerable overlap in biochemical and histological features. In addition, the histopathological features are dynamic and vary with age [3]. Therefore, making a histological diagnosis for cholestasis in infants has always been considered a challenge for many pathologists. Liver biopsy (LB) had been used in the past to diagnose specific conditions such as alpha-1 AT deficiency and Alagille syndrome. However, in the era of advanced molecular genetics and other noninvasive methods, including advanced imaging techniques and sophisticated biochemical assays, clinicians have shifted away from LB, as several pediatric liver diseases have become identifiable without the need for LB.

This revolution in the diagnosis of IC led several research groups from different countries to call for a re-evaluation of the role and indications of LB in the diagnostic algorithm of IC [4–7]. In practice when evaluating an infant with cholestasis, the main purpose for LB is to define whether BA is present or not [1, 2]. Previous studies reported that LB had an accuracy ranging between 60 and 95% in diagnosing BA [8–12]. BA, a sporadic condition, is an uncommon cause of IC in Saudi Arabia (4.7%) [13], as compared to 20–40% of IC cases in many countries around the world [14]. Data from other countries may be relevant to Arabs, however data cannot be directly extrapolated. There are several regional ethnic, genetic, metabolic or cultural differences in this part of the world that make us think that the role LB need to be re-evaluated when approaching an infant with cholestasis. For these reasons, we conducted this study to determine to what extent LB procedure has helped us in the diagnosis and management of cases with IC.

## Methods

### Study design and setting

King Fahad Medical City is one of the largest tertiary referral center for children with liver disorders in Saudi Arabia. We retrospectively reviewed our database of cholestasis cases that presented to our center in Riyadh city, the capital of Saudi Arabia, during the period from 2007 until 2019. Cholestasis was defined clinically as presence of jaundice, acholic stools, and / or itching, and biochemically when conjugated bilirubin exceeds 20 µmol/l. If there was no jaundice, we required simultaneous elevation of serum GGT and total bile acids (TBA) > 10 (Normal, 0–10 µmol/L).

### Study Population

During the study period, 533 cases of IC were referred to our center for evaluation. All infants who underwent percutaneous LB as part of diagnostic work up for cholestasis during the study period were enrolled. All infants presenting to our center with cholestasis undergo extensive work up to exclude infectious, structural, metabolic, endocrine, infiltrative and familial causes by following a diagnostic algorithm very similar to the guidelines endorsed by the North American Society of Pediatric Gastroenterology, Hepatology  and Nutrition (NASPGHAN) [2]. In our protocol, we used to consider LB in two main clinical scenarios: (1) highly suspected BA (high GGT cholestasis and pale stool); (2) when initial extensive investigations revealed no diagnosis. We excluded cases with: (1) inadequate LB specimen (when the liver tissue contained less than 5 portal spaces); (2) missing clinical information; (3) wedge biopsies obtained during IOC and Kassai portoenterostomy. Wedge biopsy was excluded because it could bias the pathologists towards a diagnosis of BA.

### Data collection

Medical records were reviewed to collect: demographics, clinical characteristics, age at presentation, date and indication of LB, complications following LB, laboratory investigations at time of LB:serum total and direct bilirubin (STB/DB), alanine transaminase (ALT), aspartate transaminase (AST), international normalization ratio (INR), GGT, and TBA, imaging findings, result of IOC, histopathological findings, the diagnosis suggested by the pathologist, and the final diagnosis. In this study, the diagnoses of all hereditary causes were confirmed by performing either direct sequencing for selected genes based on the phenotype of the patient or next generation sequencing (NGS) panels in liver diseases that incorporate a larger number of genes. In case NGS did not achieve any molecular diagnosis, the analysis was extended to whole exome sequencing.

### Liver Biopsy Procedure

Liver specimens were obtained through a percutaneous ultrasound guided liver needle under general anesthesia after correction of coagulopathy and signing informed consent. From 2007 to 2008, pediatric gastroenterologists did the LB; from 2009 until present time, a pediatric interventional radiologist has done the LB. A menghini needle gauge 18 was used to obtain a single liver tissue core. In few occasions, when we suspected mitochondrial hepatopathy, we frozen 2 mm of the liver core in liquid nitrogen immediately after collection, stored at -80 °C, and shipped on dry ice for mitochondrial studies. Following LB, patients were observed closely in hospital for vital signs, and for development of immediate complications. We do a complete blood count 6 h after LB to monitor for any hemoglobin drop.

### Liver histopathology

**Fig. 1:**
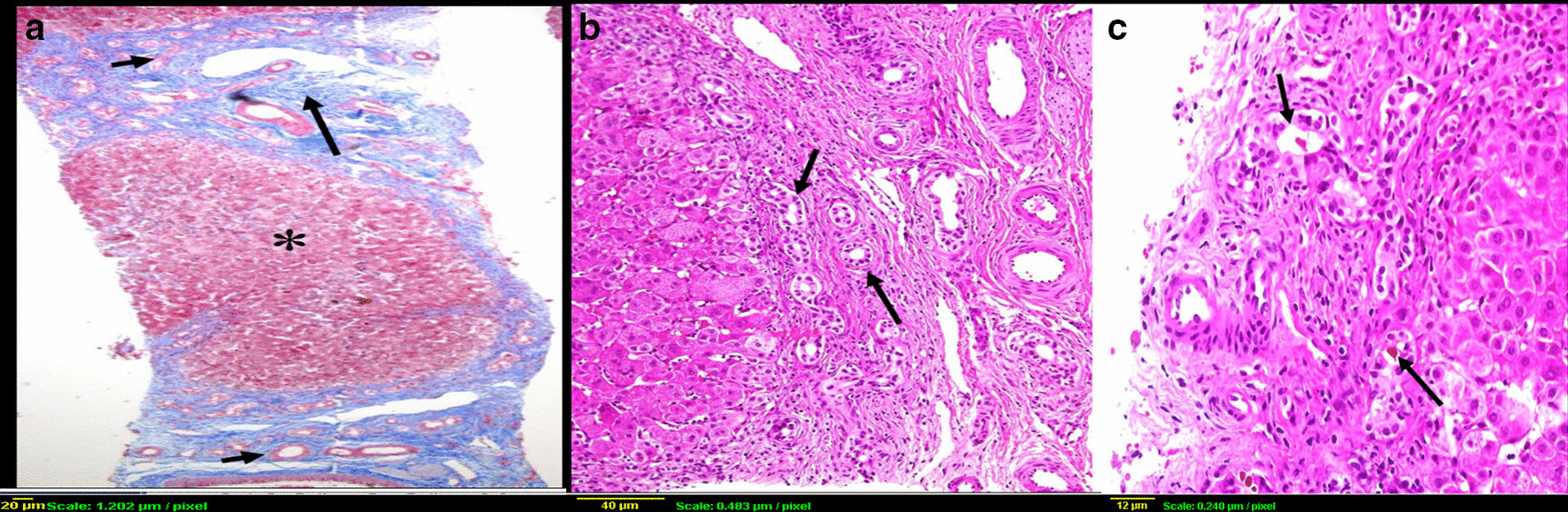
Percutaneous liver biopsy demonstrating: **a** Florid bile ductular proliferation (short arrows) and expansion of portal tract with fibrosis (long arrow), and regenerative nodule (asterisk), [Trichrome stain, original magnification ×4]; **b** Florid bile ductular proliferation [hematoxylin and eosin (HE), original magnification ×10); **c** Bile plugs in bile ductules (arrows) [HE, original magnification ×20)

**Fig. 2:**
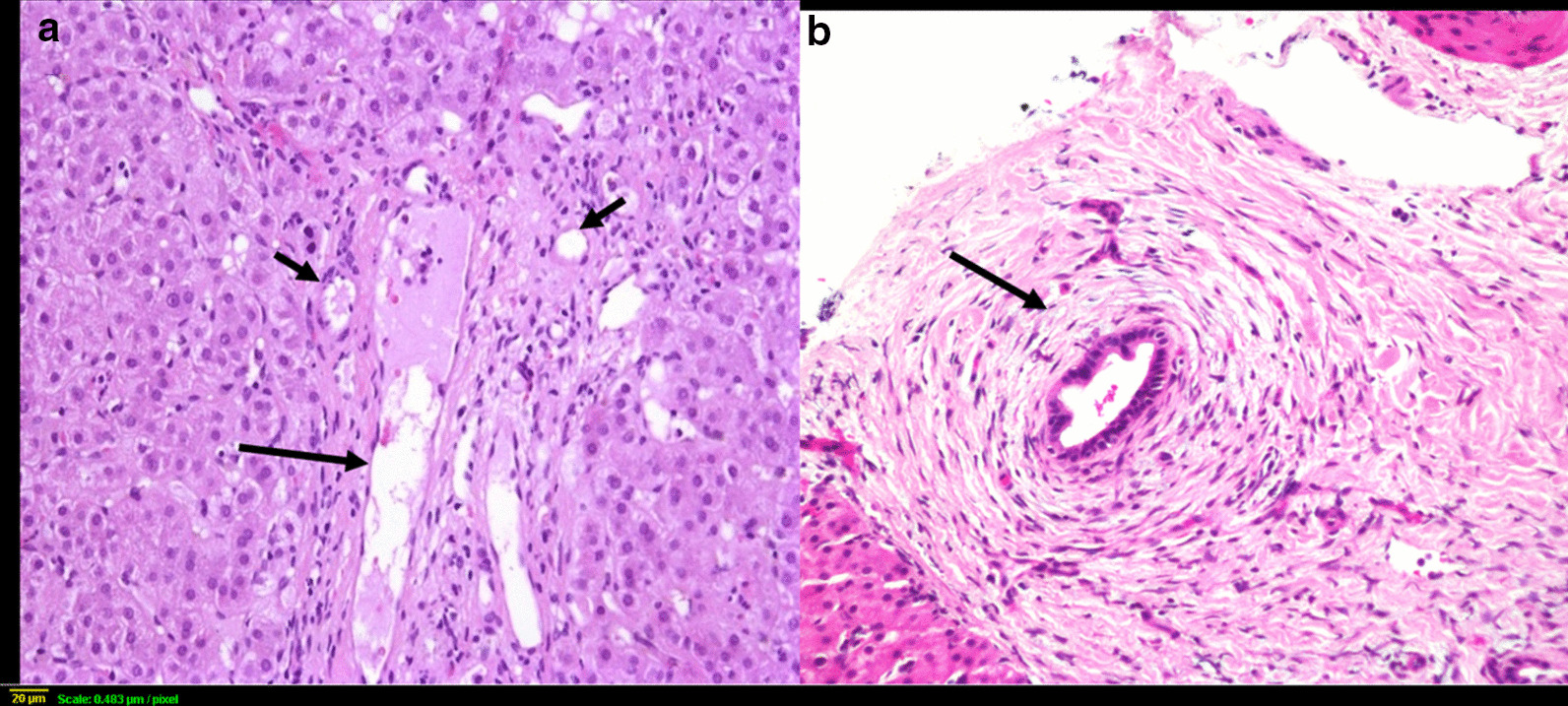
**a** Portal tract showing portal vein (long arrow), and arterioles (short arrows) and lack bile ducts in a case with Alagille syndrome (HE ×10); **b** Periductular fibrosis and onion-skin appearance (arrow) in a neonate with sclerosing cholangitis (HE ×20)

The liver specimens were fixed in 10% buffered formaldehyde paraffin-embedded, and stained with hematoxylin and eosin, Masson’s trichrome stain for fibrous tissue, and Perls’ method for iron, reticulin, and periodic acid–Schiff diastase. A single pathologist certified in gastro-hepatopathology reviewed all liver histopathology slides. The biopsy materials were screened for adequacy of size and number of portal tracts. The pathologist evaluated each liver tissue for the presence of the followings: lobular disarray, giant cell transformation, hepatocytes swelling, bile duct proliferation (mild-moderate or severe), bile duct plugs (Fig. 1b, c), bile duct paucity (Fig. 2a), bile duct injury, periductular fibrosis (onion skinning) (Fig. 2b), peri-sinusoidal fibrosis, portal fibrosis (grade I–I/grade III–IV) (Fig. 1a), canalicular cholestasis and fatty infiltration (micro- or macro-steatosis). Immunohistochemistry for bile salt export protein (BSEP) to diagnose progressive familial intrahepatic cholestasis type 2 (PFIC2), and for multidrug resistance3 protein (MDR3) to diagnose PFIC3, was not possible.

### Study outcomes

To determine the yield of LB, we categorized the yield into: (1) defined specific diagnosis and led to a specific management (specific treatment or requesting specific diagnostic investigations); (2) excluded an important diagnosis. To ascertain the accuracy of LB in arriving at a confident diagnosis or exclusion of BA in cases with high suspicion, we only accepted cases in which the pathologist reported a clear-cut histologic description “consistent with “or “not consistent” with BA. To avoid bias in estimating the diagnostic accuracy of LB, we excluded 8 wedge biopsies (out of 30). These wedge biopsies were done intraoperatively during Kasai surgery and the pathologist was not blind to diagnosis of BA.

### Definitions

- Idiopathic neonatal hepatitis (INH) was defined as a syndrome where extensive investigations into infectious, structural, metabolic, endocrine, infiltrative, and familial causes of cholestasis failed to provide an explanation.
- Diagnosis of BA was made when IOC failed to show a patent biliary tree or, when IOC was not possible, demonstration of an atretic extrahepatic biliary tree intra-operatively at Kasai surgery or liver transplant.
- Bile duct paucity was defined as reduction of bile duct profile equal or more than 40%.
- Bile duct proliferation was defined as increased number of bile ducts in the portal tract with angulated appearance.

### Statistical analysis

The data analysis includes descriptive statistics of means with standard deviations, medians, and ranges. Categorical variables were presented as numbers and percentages. Non-parametric tests were used when data were skewed and normality of data was checked by the Kolmogorov Smirnov test. Chi-square / Fisher’s exact test was used according to whether the cell expected frequency is smaller than 5, and it was applied to determine the significant association between categorical variables. The odds ratio was calculated to determine the potential risk factor that predicts diagnosis of BA. A P-value of less than 0.05 was considered as statistically significant. All data were entered and analyzed through statistical package SPSS 25 (SPSS Inc., Chicago, IL, USA).

## Results

**Fig. 3:**
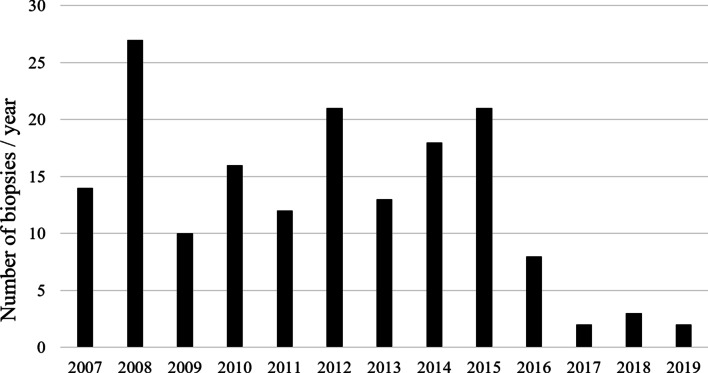
Number of liver biopsies per year

**Fig. 4:**
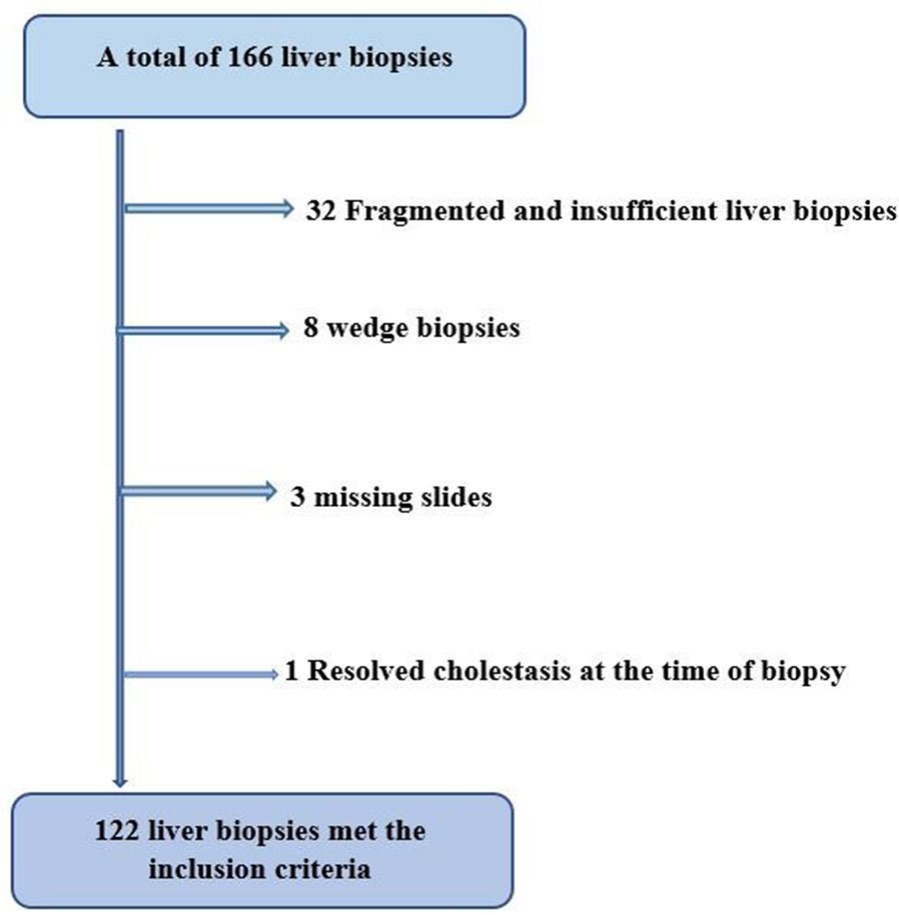
Flowchart demonstrating included and excluded liver biopsies

A total of 166 liver biopsies were obtained from 166 patients referred to our center during the study period for work up of IC (out of 533 patients; 31%). The number of LB per year is shown in Fig. 3. Among the 166 biopsies, 12 percutaneous needle biopsies (7.2%) were done by gastroenterologists early in the study period (2007–2009), 132 (79.5%) were performed by interventional radiologists under ultrasound guidance after 2009, 18 wedge LBs (11%) were done intra-operatively at the time of an intra-operative cholangiogram with Kasai procedure and if there was uncorrectable coagulopathy, and 4 LBs (2.4) were done outside our hospital but the specimens and slides were transferred to us for reading. Forty-four biopsies were excluded as detailed in Fig. 4. Eight wedge biopsies, obtained during IOC without prior percutaneous LB, were excluded from the main outcome analyses to avoid bias.Thus, 122 LB specimens met the inclusion criteria and were enrolled to analyze for the main study outcomes.

### General characteristics of the 122 patients

Demographic and baseline laboratory data of the 122 cholestatic infants (males = 77, 63%) are shown in Table 1. The mean age at onset of jaundice was 20.34 days (SD ± 43 days), and the mean age at LB procedure was 136 days (SD ± 158 days). The majority of LBs (84/122, 69%) were performed in the first 3 months of life and the remaining (38/122, 31%) were done after 3 months of life. The patients with BA had LB at a mean age of 78 days (SD ± 38 days). Pale stool and high GGT were the only 2 variables that were significantly associated with BA than non-BA group [*P* value < 0.001] (Table 1).

**Table 1:** Comparison of clinical and liver chemistry profile of BA versus Non-BA patients

Variables	BA group (n = 22)	Non-BA group (n = 100)	*P* value
Gender male:female	1.3	1.8	0.172
Birth weight (kg) ± SD	2.83 (± 0.40)	2.74 (± 0.64)	0.496
Gestational age (preterm)	3 (10%)	10 (10%)	0.831
Mean Age at onset of jaundice (days) ± SD	5.53 (± 8.41)	23.79 (± 48.57)	0.059
Mean Age at biopsy (days) ± SD	78.76 (± 38.04)	150.75 (± 184.86)	0.051
Pale stool	22 (100%)	42 (42%)	< 0.001
Hepatomegaly	16 (72%)	65 (65%)	0.696
Labs (Mean ± SD)			
STB (µmol/L)	189.97 (± 64.43)	156.26 (± 91.14)	0.049
Direct bilirubin (µmol/L)	156.13 (± 51.77)	127.32 (± 71.50)	0.056
ALT (U/L)	244.88 (± 144.85)	260.64 (± 283.33)	0.787
AST (U/L)	378.72 (± 250.19)	348.77 (± 282.97)	0.624
GGT (U/L)	640.88 (± 507.79)	308.35 (± 392.31)	< 0.001
Alkaline phosphatase (U/L)	803.8 (± 416.43)	746.98 (± 465.23)	0.572

*kg* kilogram, *STB* serum total bilirubin, *ALT* Alanine aminotransferase, *AST*Aspartate aminotransferase, *GGT* Gamma-glutamyl transferase/transpeptidase

### Differential diagnosis of the 122 IC cases

**Fig. 5:**
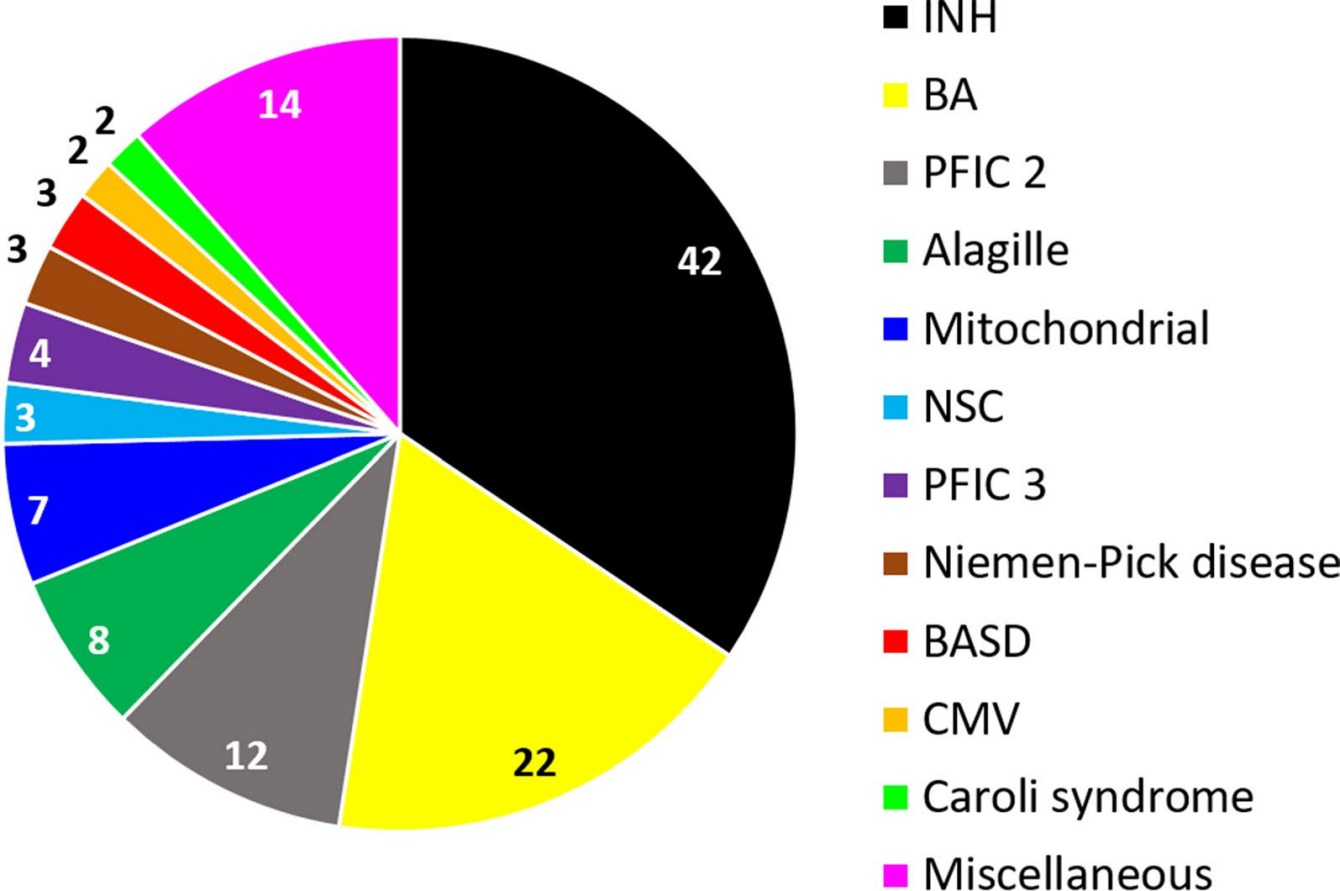
Differential diagnosis of liver biopsy driven infantile cholestasis. *INH* ideopathic neonatal hepatitis; *BA* biliary atresia; *PFIC* progressive familial intrahepatic cholestasis; *NSC* neonatal sclerosing cholangitis; *BASD* bile acid synthesis disorder; *CMV* cytomegalovirus. Miscellaneous causes include, one case each: Dubin-Johnson syndrome, Tight junction (TJP) mutation (USP53), Multifactorial: prematurity and total parenteral nutrition (TPN), Spino-cerebellar ataxia type XXI, Down syndrome, Progressive familial intrahepatic cholestasis 1 (PFIC 1), Bile duct paucity (FOXA1 gene mutation), Gaucher disease, Ciliopathy WDR 19 mutation, Ciliopathy TTC 26 gene mutation, Hepatitis B virus cholestasis (HBV), Panhypopituitarism, Zellweger syndrome, IARS mutation = Infantile hepatopathy steatosis and portal-tract fibrosis, intellectual disability, muscular hypotonia and growth retardation syndrome

The final diagnosis of the 122 cases with IC is shown in Fig. 5. Overall, the most common diagnosis was INH (n = 42; 34%), followed by BA (n = 22; 18%), PFIC 2 (n = 12; 10%), Alagille syndrome (n = 8; 6.5%), mitochondrial hepatopathy in 7 cases (5.7%), and miscellaneous causes represents 11.5% (n = 14) of cases.

### The sensitivity and specificity of LB in diagnosis of BA

The main indication for LB was high suspicion of BA, in 46 cases (38%). In the remaining 76 cases (62%), LB was performed as part of diagnostic work up because initial investigations revealed no diagnosis. Among the 46 cases with high suspicion of BA, 22 cases were finally confirmed to have BA: 19 of the 22 cases (86.4%) were truly diagnosed histologically as BA (true positive) and 3 were mislabeled as non-BA (false Negative). Of the remaining 24 non-BA cases, non-BA was identified correctly on histology in 16 cases (true negative) but 8 cases were mislabeled as BA (false positive), resulting in a sensitivity of 86.4%, specificity (66.7%), PPV (70.4%), NPV (84.2%), and diagnostic accuracy 76%. Table 2 outlines in details the reasons for the discrepant assignment of 3 clinically proven BA cases to the category “not consistent with BA”. The diagnoses of the eight cases mislabeled as BA were as following: INH in 5, and neonatal sclerosing cholangitis, Caroli disease, and Alagille, one for each. IOC demonstrated patency of the common bile duct and excluded the diagnosis of BA in all of them.

**Table 2:** The histological description of the 3 confirmed biliary atresia cases mislabeled as non-BA

	Age at LB (days)	Histology on percutaneous LB	Wedge LB	US liver	MRI liver	IOC
1	60	No significant BD proliferation. No BD plugs. BD injury with few vanished BD. Moderate portal fibrosis. Canalicular cholestasis. Pathologist suggested neonatal sclerosing cholangitis	Marked BD proliferation. BD plugs. BD injury not seen. Fibrous septae and marked portal fibrosis	CBD visualized. Normal GB size	Hypoplastic GB	Failed due to atrophic GB
2	90	Mild BD proliferation. No BD plugs. Marked portal fibrosis with septae. Hepatocytes swelling. GC transformation. Canalicular cholestasis. Pathologist suggested BASD and PFIC 2	ND	CBD non-visualized Echogenic cord sign GB non-visualized	CBD non-visualized GB non-visualized	ND*
3^‡^	60	Mild BD proliferation. No BD plugs. Marked portal fibrosis with septae. Hepatocytes ballooning. Canalicular cholestasis. Pathologist suggested BASD and PFIC 3	ND	CBD non-visualized GB atrophic	CBD non-visualized GB non-visualized	ND*

*LB* liver biopsy, *US* ultrasound, *MRI* magnetic resonance image, *IOC* intraoperative cholangiogram, *BD* bile ducts, *CBD* common bile duct, *GB* gall bladder, *GC* giant cell, *BASD* bile acid synthesis disorder, *PFIC* progressive familial intrahepatic cholestasis, *ND* not done, ND* not done because of late presentation, the child underwent liver transplantation and biliary atresia was confirmed intraoperatively

^‡^Patient has heterotaxy syndrome, intrahepatic interruption of inferior vena cava, hemi-azygous continuation, and the superior mesenteric vein was seen anterior to the superior mesenteric artery

### Comparison of BA histopathology versus other etiologies

Overall, there were 22 BA and 100 non-BA cases (Table 3). Several histological features were observed mainly in BA group and found to be significant predictors of BA: marked bile ductular proliferation, bile duct plugs, portal fibrosis, and canalicular cholestasis (Fig. 1a–c). Bile duct plugs and marked bile ductular proliferation were the strongest predictors of BA (OR 48, *P* value < 0.001; OR 8.699, *P* value < 0.001, respectively), across the major etiologies subgroups. Conversely, three features were not existing in any BA case: lobular disarray, bile duct paucity, perisinosoidal fibrosis, and fatty infiltration. Bile duct paucity was most significantly associated with the non-BA group of cases. Most of the other features had a similar distribution between BA and non-BA cases. Within the mitochondrial group, fatty infiltration (micro- and / or macrosteatosis) was strong predictor of the diagnosis (72% versus 0 in BA; *P* < 0.001). Within the PFIC2 group, prominent hepatocytes swelling (91% versus 22.72% in BA; *P* = 0.002), lobular disarray (27% versus 0 in BA; *P* = 0.004) and marked giant cell transformation (82% versus 18.18% in BA; *P* < 0.001) distinguished cases of PFIC2 from the BA cases.

**Table 3:** Comparison of histopathopathological features in BA group versus Non-BA patients

Histopathology variables	BA group (n = 22)	All Non- BA group (n = 100)	OR[95% C.I]	*P* Value
Lobular disarray no. (%)	0	11 (11)	–	0.103
Mild-mod bile duct proliferation no. (%)	14 (63.63)	46 (46)	2.054 [0.792–5.331]	0.134
Marked bile duct proliferation no. (%)	8 (36.36)	8 (8)	6.571 [2.123–20.34]	< 0.001*
Bile duct paucity/loss no. (%)	0	23 (23)	–	0.013*
Bile duct injury no. (%)	4 (18.18)	15 (15)	1.259 [0.374–4.242]	0.709
Periductular fibrosis no. (%)	2 (9.09)	5 (5)	1.9 [0.344–10.497]	0.455
Perisinosoidal fibrosis no. (%)	0	11 (11)	–	0.103
Portal fibrosis no. (%)	22 (100)	83 (83)	–	0.037*
Giant cell formation no. (%)	4 (18.18)	23 (23)	0.744 [0.229–2.419]	0.622
Hepatocyte swelling no. (%)	5(22.72)	52 (52)	0.271 [0.093–0.793]	0.013*
Canalicular cholestasis no. (%)	22 (100)	73 (73)	–	0.006*
Bile duct plugs no. (%)	10 (45.45)	3 (3)	26.944 [6.494–111.788]	< 0.001*
Fatty infiltration no. (%)	0	11 (11)	–	0.103

*p-value is statistically significant (< 0.05)

Paucity of interlobular bile duct was present in 3 of the 8 Alagille syndrome cases; they had LB procedure done at median age 5 months (range 4–12 months) (Fig. 2a). The LB in the remaining 5 patients, done at median age 2.5 months (range 1.5–4 months), manifested mild-moderate bile duct proliferation and the pathologist raised possibilities of BA, neonatal sclerosing cholangitis, and PFIC3. Table 4 list the other causes of bile duct paucity in our cohort.

**Table 4:** Causes of bile duct paucity

Diagnosis	Number of cases
INH	9
USP53 Gene mutation (TJP mutation)	1
PFIC 2	4
Alagille syndrome	3
Down syndrome	1
CMV	1
FOXA1 gene mutation*	1
Biliary atresia	1
TPN-associated cholestasis	1
HBV	1
Panhypopituitarism	1

*INH* ideopathic neonatal hepatitis, *TJP* tight junction protein, *PFIC* progressive familial intrahepatic cholestasis, *CMV* cytomegalovirus, *TPN* total parenteral nutrition, *HBV* hepatitis B virus

*FOXA1 gene mutation, c.313G > A, (p.Gly105Ser), is not associated with human disease, however FOXA1 gene is a critical transcription factor for normal bile duct development (Li 2009, J Clin Invest 119: 1537–45), Homozygous mutation: The patient presented at 2 weeks with low GGT cholestasis

Periductular fibrosis with onion-skin appearance was a feature in the 3 cases with probable neonatal sclerosing cholangitis (Fig. 2b), but was also seen rarely with other etiologies (BA = 2, Alagille syndrome = 1). Micro- and / or macrosteatosis was prominent in 3 conditions: mitochondrial hepatopathy (5/7 cases; 72%), Niemann-Pick disease (3/3 cases; 100%), and 2 idiopathic cases.

### Study outcomes

Analysis of the data of the 122 cases for the study outcomes showed that LB had a direct impact on clinical management in 52 cases (42.6%):

1. The true diagnosis was suggested by LB in 36 cases [BA = 19; mitochondrial disease = 5; sclerosing cholangitis = 3; Alagille syndrome = 3; PFIC2 and CMV infection = 2 cases, each; Niemann-Pick disease and PFIC3, one case, each]. The histologic description “consistent with BA” in the 19 cases led to IOC that confirmed BA diagnosis, while the histologic description that suggested a hereditary disease has guided the diagnostic process and focused the molecular genetic testing. Diagnosis of CMV infection in two cases led to initiation of ganciclovir therapy.
2. LB excluded BA and avoided IOC in 16 cases with high suspicion of BA.

On further sub-analysis among the 46 highly suspected BA cases, LB diagnosed BA in 19, ruled out BA in 16 cases, and suggested the true diagnosis in 4 cases (NSC = 3, CMV = one case), resulting in an overall positive impact on diagnosis and management in 39 of the 46 cases (85%). Among the remaining 76 cases with low suspicion of BA, LB suggested the true diagnosis or helped to initiate a specific management in only 8 cases (10.5%). In contrast, gene testing (target gene test, cholestasis panel, or whole exome sequencing) confirmed the diagnosis in 48 of the 76 cases (63%).

No complications occurred following all the LB procedures.

## Discussion

This report is the first from Arabs and the largest from a single center to evaluate the impact of the LB on diagnosis and management of IC, and highlights a number of important observations. First, the LB had a high yield in the diagnosis and management of IC cases when used as a tool in the work up of IC cases with high clinical suspicion of BA (85%), mainly by confirming or excluding BA diagnosis; in contrast, the LB had a low diagnostic yield and rarely changed the management and the work up of IC cases with a low suspicion of BA (10%). Second, on the contrary, the use of various forms of molecular gene testing in the group with a low suspicion of BA revealed diagnosis in 63% of the cases. Third, LB had a sensitivity of 86.4% and specificity of 66.7% in diagnosing BA with bile duct plugs and marked bile ducts proliferation were the strongest predictors of BA diagnosis. An important finding from our data is that the presence of micro- or macrovesicular steatosis can assist in narrowing the range of diagnostic tests that need to be performed toward metabolic liver diseases in particular mitochondrial hepatopathy and storage diseases. Bile duct paucity though it features Alagille syndrome, however may not be present in infants less than 4 months of age; hence, the LB timing is crucial in histological interpretation.

In practice many centers use LB mainly to define whether BA is present or not in order to facilitate timely Kasai surgery, as early surgery for BA before 8 weeks is one of the most important prognostic factors of successful outcome [1, 2]. In our cohort, the average age at which the LB was done to rule in or rule out BA was about 11 weeks. This late timing of the LB is a consequence of late referral to our center at a median age of 65 days [13]. As a result, the outcome of BA cases in our center was poor with a success rate of KPE at 33% and the short-term survival rate without LT at 14.3% [13], as compared to data from Western countries and Japan which showed a success rate of KPE at 60 to 80%, and 4-year native liver survival of 40 to 60% [15–18]. Many of the liver diseases in infants manifest cholestasis with significant overlap clinically and biochemically which makes early diagnosis of BA a clinical challenge. The serum matrix metalloproteinase-7 (MMP-7) is a promising noninvasive biomarker that at high concentration has high diagnostic accuracy in differentiating BA from other etiologies of IC, however its clinical application requires further standardization and testing in future studies with larger cohort sizes and different ethnicities [19, 20]. Careful selection of cases with a high index of suspicion of BA to undergo LB would improve the diagnostic yield of the LB, facilitate early diagnosis of BA, and avoid un-necessary invasive procedure in cases with low suspicion of BA. In a baby with cholestasis, the combination of pale (acholic) stool and high GGT are valuable clinical clues of BA as observed in our study. Other clinical and radiologic clues of BA include the presence of extra-hepatic anomalies such as heterotaxy or asplenia/polysplenia, the triangular cord sign at porta-hepatis, and hypoplastic or atrophic gallbladder [2]. In contrast, findings such as pigmented stools and normal or low GGT make the possibility of BA very low, and the LB when performed is less likely to add substantial diagnostic information.

After clinician carefully select IC cases with high clinical suspicion of BA to undergo LB, here it comes the role of pathologist to identify histopathological features that provide important clues to the correct diagnosis of biliary obstruction. In our study several histopathological features of BA were shared with other non-BA etiologies, however, we found that bile duct plugs and marked ductular proliferation were strong independent histologic predictors of BA. When these are found in a LB histology in association with portal fibrosis, absent or rare hepatocellular giant cell transformation and no lobular disarray or sinusoidal fibrosis, the diagnosis of BA is most probable and should prompt confirmation by IOC. Other investigators demonstrated additional histological features that when found in combination with the above-mentioned histological features, favor BA diagnosis. These include portal stromal edema and absent or rare extramedullary hematopoiesis [12, 21]. In our study, apart from portal fibrosis and canalicular cholestasis which were commonly shared by 83% and 73% of the 100 non-BA cases respectively, no single histological feature was consistently present in all BA cases which could make an accurate histological diagnosis of BA a challenge for many pathologists. Marked bile duct proliferation which is generally regarded as a key feature of biliary obstruction, was absent in as many as 63% of our BA cases, and bile ductular plugs were absent in 54% of BA cases, which could be explained by the variability in the severity of the histologic features and timing of LB. Three cases of BA were missed (3/22, 13.6%); in all of them, there were mild or no proliferation of bile ductules and no bile duct plugs which deterred the pathologist from considering BA diagnosis. Our pathologist correctly identified BA in 86% of the confirmed BA cases, which is similar to pathologists that participated in the multicenter Biliary Atresia Research Consortium on histological assessment of BA who identified signs of biliary obstruction in 89% (range 79–98%) of clinically proven cases of BA [21]. Overall, the reported accuracy rate in several previous studies ranged between 60 and 95% [9–12]. This variability in BA diagnosis could be attributed to several factors like the pathologists’ experience and timing of LB relative to the course of the disease, particularly when done early in the disease process (< 6 weeks’ age), because progression of histologic features evolves with age [1–3]. Having said that, in a multicenter large study 10 out of 11 BA cases less than 30 days of age at biopsy were correctly assigned to the category “consistent with large duct obstruction” [21]. This suggests that though the histologic progression in BA might not follow the same time course in every patient, and thus age at the time of biopsy should not by itself preclude the appropriate diagnosis, especially in experienced pathologist’s hands. In our study, we think that the diagnostic performance of LB could have been better if the wedge biopsies were not excluded. One of the 3 BA cases that were read as “non-consistent with BA” underwent wedge biopsy at the time of IOC which showed the characteristic histopathological features of BA. In a multicenter study where 14 BA cases were falsely thought to be “not consistent with BA” on percutaneous LB, the corresponding wedge LB in 9 of these cases demonstrated features of large duct obstruction [12]. The authors suggested that the wedge biopsies were more representative, as some of the characteristic features are better noted in larger portal tracts not sampled by the needle biopsy.

The commonest cause of IC worldwide is BA, ranging between 20 to 40% [14]. In contrast, BA is an uncommon cause of IC in Saudi Arabia (5%) [13]. Our study cohort represents a LB-driven differential diagnosis of IC in our community as it is composed of a highly selective group of infants who underwent LB due to high suspicion of BA or uncertainty of diagnosis following unrevealing investigations. As a result, selection bias caused overrepresentation of BA (18%) and INH (34%). On the other hand, out of 533 cases of IC presenting to our hospital during the study period, familial cholestasis comprises the commonest cause of IC in Saudi Arabia (24%, unpublished data). Other hereditary etiologies (e.g. Metabolic/Storage disease and Mitochondrial hepatopathy) contributed to 15%, hence all together 39% of the IC cases could have been diagnosed by non-invasive diagnostic means other than LB. The availability of emerging diagnostic technologies including enzyme analysis, biochemical assays, DNA sequencing, and advanced imaging modalities has challenged the role of LB in the diagnostic work up of an infant with cholestasis, particularly if BA is not a clinical suspicion (i.e. when serum GGT levels are low/normal or stool is clearly pigmented). Nowadays there are several commercially available cholestasis gene “panel”/chip which include many of the common mutations of inherited syndromes of cholestasis, that are becoming increasingly affordable at a lower cost [22–25]. Incorporation of gene panels and whole exome sequencing in evaluation of IC in place of LB in the presence of proper clinical context of consanguinity, similar family disease, and a low suspicion of BA is expected to have better diagnostic yield and avoid un-necessary invasive procedures. In line of this approach, the number of LB has reduced over time from 27/year in 2008 to 2–3/year in the last 3 years (Fig. 3).

Besides the retrospective design of the study and the potential selection bias mentioned above, lack of complementary techniques such as immunohistochemical methods might have contributed to the low diagnostic yield of LB in cases with low suspicion of BA. Immunohistochemistry for biliary transporters like BSEP in PFIC2 and MDR3 in PFIC3, is helpful to facilitate orientation of specific genes testing. The rate of insufficient LB specimens was relatively high in our center which could have been due to 2 reasons. First, some of these insufficient specimens were obtained early in the study period during which our trainees went through a learning curve to achieve procedure skills; second the late timing of LB in our study cohort means more chance to find advanced fibrosis which might explain several fragmented LB specimens.

## Conclusions

Our results indicated that LB continues to be a safe and important tool in the diagnostic work up of cases with high suspicion of BA. However, the role of LB needs to be re-evaluated in infants with low suspicion of BA in whom early incorporation of cholestasis next-generation sequencing panels in place of LB can have better diagnostic yield.

## Data sharing statement

The datasets used and/or analysed during the current study available from the corresponding author on reasonable request.

## Abbreviations

LB: liver biopsy
IC: infantile cholestasis
BA: biliary atresia
GGT: gamma-glutamyl transferase
PPV: positive predictive value
NPV: negative predictive value
IOC: intraoperative cholangiogram
STB: Serum total bilirubin
DB: direct bilirubin
TBA: total bile acids
ALT: alanine transaminase
AST: aspartate transaminase
NGS: Next generation sequencing
INR: international normalized ratio
PFIC: progressive familial intrahepatic cholestasis
MDR: multidrug resistance
INH: idiopathic neonatal hepatitis
CMV: cytomegalovirus hepatitis
BSEP: bile salt export pump
